# Prospective study of serum 25(OH)-vitamin D concentration and risk of oesophageal and gastric cancers

**DOI:** 10.1038/sj.bjc.6603834

**Published:** 2007-06-05

**Authors:** W Chen, S M Dawsey, Y-L Qiao, S D Mark, Z-W Dong, P R Taylor, P Zhao, C C Abnet

**Affiliations:** 1 Department of Epidemiology, Cancer Institute (Hospital), Chinese Academy of Medical Sciences, Beijing, People's Republic of China; 2 Nutritional Epidemiology Branch, Division of Cancer Epidemiology and Genetics, National Cancer Institute, Bethesda, MD, USA; 3 Departments of Preventive Medicine & Biometrics, University of Colorado Health Sciences Center, Denver, CO, USA; 4 Genetics Branch, Division of Cancer Epidemiology and Genetics, National Cancer Institute, Bethesda, MD, USA

**Keywords:** vitamin D, oesophageal cancer, gastric cancer, cohort study, China

## Abstract

We prospectively examined the relation between pretrial serum vitamin D status and risk of oesophageal and gastric cancers among subjects who developed cancer over 5.25 years of follow-up, including 545 oesophageal squamous cell carcinomas (ESCC), 353 gastric cardia adenocarcinomas, 81 gastric noncardia adenocarcinomas, and an age- and sex-stratified random sample of 1105 subjects. The distribution of serum 25(OH)D was calculated using the known sampling weights. For the cohort as a whole, the 25th, 50th, and 75th percentile concentrations of 25(OH)-vitamin D were 19.6, 31.9, and 48.7 nmol l^−1^, respectively, and we found that higher serum 25(OH)D concentrations were associated with monotonically increasing risk of ESCC in men, but not in women. Comparing men in the fourth quartile of serum 25(OH)D concentrations to those in the first, we found a hazard ratio (HR) (95% confidence interval (CI)) of 1.77 (1.16–2.70), *P* trend=0.0033. The same comparison in women had a HR (95% CI) of 1.06 (0.71–1.59), *P* trend=0.70. We found no associations for gastric cardia or noncardia adenocarcinoma. Among subjects with low vitamin D status, higher serum 25(OH)D concentrations were associated with significantly increased risk of ESCC in men, but not in women. Further refinements of the analysis did not suggest any factors, which could explain this unexpected result.

Exposure of the skin to type-B ultraviolet radiation leads to the production of vitamin D. Fortified dairy products, eggs, fish, liver, and some plant foods also provide vitamin D. Three studies that used different methods for estimating vitamin D exposure showed that conditions that should favour higher vitamin D status were associated with lower risk of oesophageal or stomach cancer. Specifically, higher potential solar radiation in lower latitudes (Grant, 2002), higher vitamin D intake (Launoy *et al*, 1998), and higher vitamin D exposure index (Giovannucci *et al*, 2006) were associated with reduced risk of oesophageal and/or stomach cancer. One hypothesis for this protection is that vitamin D can suppress diet-induced epithelial cell hyperproliferation (Xue *et al*, 1999).

Vitamin D status can be assessed directly using serum 25-hydroxy vitamin D (25(OH)-D) concentration, the best indicator of status (Zittermann, 2003). While not the active form, it can be converted into the active 1,25(OH)_2_ vitamin D in the kidney and at other sites. It is not known whether the oesophagus or stomach expresses the 25(OH)D-1*α*-hydroxylase enzyme that is necessary for extrarenal activation (Townsend *et al*, 2005).

Linxian, a semi-arid mountainous area in central China (36° N latitude), has a mainly rural population with some of the highest rates of oesophageal squamous cell carcinoma (ESCC) and gastric cardia adenocarcinoma in the world, with age-standardised rates for both men and women of about 100/100 000 year^−1^ for the two tumours combined (Ke, 2002). Prospective studies in Linxian showed increased risk of these cancers in subjects with lower concentrations of serum selenium (Mark *et al*, 2000), serum *α*-tocopherol (Taylor *et al*, 2003), and tissue zinc (Abnet *et al*, 2005). Deficiencies of *β*-carotene, *β*-cryptoxanthin, and retinol are common but not associated with increased risk of these tumours (Abnet *et al*, 2003). No previous reports on vitamin D status exist for Linxian where the traditional diet provides little vitamin D because fatty fish and liver are taken sparingly (Zou *et al*, 2002), median egg intake was only 10 per year (Tran *et al*, 2004), and fortified products are not available.

In the current study, we have examined the relation between serum 25(OH)D and the risk of ESCC, gastric cardia adenocarcinomas, and gastric noncardia adenocarcinoma in a large population-based cohort of subjects.

## METHODS

### Cohort population

Subjects were selected from the cohort of all participants in the General Population Trial of Linxian. Elsewhere we have described in detail the design, choice of intervention agents, methods of conduct, and primary end-point analyses of the trial (Blot *et al*, 1993; Li *et al*, 1993). In brief, the participants were 29 584 healthy adults aged 40–69 years from four Linxian communes. In the spring of 1985, 1 year before the start of the intervention, each participant was interviewed, given a brief physical examination, and had 10 ml of blood drawn. Blood was stored on ice for 3–6 h during transport to the field station. The serum was separated by centrifugation, then aliquoted, and immediately stored at −45°C for 3–4 days. The serum was stored at −85°C for long-term storage after transport to Beijing on dry ice and was not thawed until used in the vitamin D assays. Intervention began in March 1986 and continued through May 1991. Subjects who died or developed cancer in the interval between recruitment and the start of the intervention were excluded from the trial and this cohort. In accord with a partial factorial design, the participants were randomly assigned to receive either a vitamin–mineral combination or a placebo. Vitamin D was not included in the General Population Trial as an intervention agent. Village doctors ascertained mortality among trial participants through monthly follow-up; loss to follow-up was essentially zero. Cancers were ascertained through local commune and county hospitals, supplemented by a study team that provided clinical and diagnostic services, including endoscopy, for patients with symptoms suggestive of oesophageal or gastric cancer. A panel of US and Chinese experts reviewed the diagnostic material for 85% of the cancer cases in this study. All oesophageal cancers were squamous cell carcinomas. There were no adenocarcinomas in the oesophagus in this study so we did not need to separate oesophageal adenocarcinomas from gastric cardia cancers. For anatomic localisation of gastric adenocarcinomas, we defined cardia cancers as those located in the most proximal 3 cm of the stomach and non-cardia gastric cancers were those that originated distal to this region. Ninety-five percent of the anatomic localisations were made with the use of endoscopy, surgery, and/or x-rays. For cancer cases without diagnostic materials and for deaths due to causes other than cancer, senior Chinese diagnosticians conducted reviews. We obtained informed consent from each participant before trial enrollment. Throughout the study, human subject protection procedures were approved by the Institutional Review Boards of the U.S. National Institutes of Health and the Chinese Academy of Medical Sciences.

### Selection of study participants for serum measurements

We used a stratified case–cohort design (Prentice, 1986; Self and Prentice, 1988) to select individuals for serum measurements from the cohort of all participants in the General Population Trial. By the end of the trial, there were 640 incident ESCC, 435 incident gastric cardia adenocarcinomas, and 104 gastric non-cardia adenocarcinomas (Blot *et al*, 1993). Overall, 83% of the case subjects had adequate serum and a successful measurement of serum vitamin D (545 oesophageal, 353 gastric cardia, and 81 gastric non-cardia cancer subjects). In addition, we measured serum concentrations in a stratified random sample of all trial participants, without regard to outcome, as the comparison group (referred to as the subcohort). The six strata were defined by sex and the following three age categories at the start of intervention: (1) 50 years old or younger, (2) older than 50–60 years old, and (3) older than 60 years. A sufficient number of cohort subjects were drawn from each stratum to achieve a ratio greater than 1 : 1 of control subjects to case subjects for incident oesophageal and gastric cardia cancers combined. Overall, we measured serum 25(OH)D concentration on 1105 subcohort subjects and 979 case subjects.

### Laboratory analysis

Serum 25(OH)D concentrations were determined using OCTEIA 25-hydroxy Vitamin D enzyme immunoassay (IDS Inc., Fountain Hills, AZ, USA) in the nutrition laboratory of the Cancer Institute, Chinese Academy of Medical Sciences. This assay has been shown to be accurate and reliable by the International Vitamin D Quality Assessment Scheme (www.deqas.org), although it may slightly overestimate serum 25(OH)D concentrations when they are in the high range (Carter *et al*, 2004). The samples were analysed in a sequence designed to minimise the possible bias in the estimation of cancer risk that could be introduced if serum measurements varied by time. Within every group of 10 samples, a sample from a case subject was always accompanied by a sample from a control subject from the same sex and age stratum. Case subjects with each of the three cancer types in each of the six strata were mixed throughout. For assessment of assay reliability, each plate had one quality control sample of pooled serum and two control serum samples, one with a high concentration and one with a low concentration. Laboratory personnel were blind to the identity of all samples. A total of 174 blinded assay reliability specimens were measured. Coefficients of variation for the pooled serum sample, high control, and low control were 16, 7, and 11%, respectively. The methods used to assay serum selenium, *α*-tocopherol, retinol, and cholesterol measurements have been described previously (Mark *et al*, 2000; Abnet *et al*, 2003; Taylor *et al*, 2003).

### Statistical analysis

To examine graphically the shape of the serum 25(OH)D distributions, we used histograms and quantile–quantile plots and found that the distribution was skewed. Log transformation improved normality. All estimates of means were made using the log-transformed values. In the tables we report log means returned to the arithmetic scale, that is geometric means. Throughout the paper, all *P*-values we report are from two-sided tests and an *α* ⩽0.05 was considered statistically significant.

The mean values and quantiles were calculated using the known sampling weights from the entire General Population Trial cohort for each individual in the study. Thus, the means and quantiles for the serum 25(OH)D concentrations given for the subcohort in Table 2 are estimates of the means and quantiles of the entire General Population Trial cohort and not, as is generally the case, the means and quantiles of those who never develop the cancers under study. We tested for serum 25(OH)D concentration differences between groups using the Wilcoxon rank–sum test.

We measured the time to cancer as time since March 1986 (start of the intervention), as opposed to time since blood collection. Individuals who died or developed cancer in this 1-year interval were excluded from the General Population Trial and this analysis. When analyzing cancers at a specific site we treated persons with cancers at other sites as censored at the time of cancer occurrence. We estimated hazard ratios (HR) and 95% confidence intervals (CI) using the case–cohort estimator for the Cox proportional hazards models available in the EPICURE software package (Hirosoft International Corp., Seattle, WA, USA). All estimates came from models stratified on the six sex–age sampling strata. Additional stratum-specific age terms for continuous age were used to adjust for variation within age stratum. Although they did not materially alter the risk estimates, we retained tobacco smoking and alcohol drinking in the models because they were specified as potential confounders *a priori*. Body mass index was tested and found not to alter risk estimates and was dropped from the final models. Nested models were compared and *P*-values calculated using score tests. To examine the proportionality assumption, we used models that allowed time-dependent relative risks and found no suggestion that HRs varied with time. We also deleted the first 2 years of follow-up and found similar HRs to the estimates using the full follow-up time.

We examined three different metrics for serum 25(OH)D concentration: (1) as a continuous variable with the units standardised to 15 nmol l^−1^, (2) as quartiles based on the distribution in the subcohort, and (3) as a trend with subjects assigned the ordinal quartile value as their exposure. We used sex-specific quartile cutpoints in the sex-stratified analyses. We tested for and found a statistically significant interaction for the association between serum 25(OH)D concentration and risk of ESCC by sex using the product of the sex and continuous serum 25(OH)D concentration. We found no significant interaction by sex for either gastric cancer subsite. But, for comparison we present estimates stratified by sex at each site. We found no statistically significant interactions by age, serum selenium concentration, or serum retinol concentration using either continuous or categorical constructs for the interacting variables. We tested for deviations from log linearity by adding quadratic terms to the continuous models and by adding separate parameters for individuals in the lowest and highest deciles (threshold effects). No statistically significant quadratic deviations or thresholds were found. We performed a sensitivity analysis to test for outliers or influential points in the continuous main-effect HRs by deleting the upper and lower 1% of serum values and there were no substantial changes in relative-risk estimates. Finally, we tested for a difference in the slope of the linear variable above and below the level of adequacy (40 nmol l^−1^) using an interaction between the continuous serum concentration and an adequacy indicator variable. There was no evidence of deviations from linearity in the association between serum concentrations and risk of cancer.

## RESULTS

Table 1 presents the characteristics of the randomly selected subcohort and the cancer cases overall and by sex. A total of 70% men smoke lightly and drink little or no alcohol, and among women, smoking was almost completely absent and alcohol consumption was also low.

Table 2 presents the serum 25(OH)D geometric means and quantiles in the population overall and stratified by sex, smoking and drinking. Women had slightly lower serum 25(OH)D concentrations than men. Men in the youngest age strata had lower serum concentrations than older men, but there were no clear age differences in women. Although the geometric means in men never and ever smokers were not significantly different (Wilcoxon rank–sum *P*=0.13), smokers had a narrower distribution that led to a higher concentration at the 10th percentile and a lower concentration at the 90th percentile. Men who drank any alcohol in the past 12 months had lower serum 25(OH)D at each quantile compared to men who did not drink any alcohol, but the overall distributions were not significantly different (Wilcoxon rank—sum, *P*=0.40). It should be noted that using this construct, any alcohol drinking in the previous 12 months is associated with a non-significantly decreased risk of ESCC in this population (Tran *et al*, 2004). Overall, serum 25(OH)D concentrations were higher in subjects who developed each of the cancers compared to the subcohort (Table 2). When examined separately by sex, the biggest difference was for men who developed ESCC. The geometric mean 25(OH)D concentration for the men in the subcohort was 33.3 nmol l^−1^ and for men who developed ESCC it was 40.1 nmol l^−1^.

We present the multivariate adjusted HRs and 95% CIs for the association between serum 25(OH)D concentration and cancer risk in Table 3. We found a significant association with ESCC, but no significant association for either gastric cancer site. We also found a significant interaction between serum 25(OH)D and sex in the association with ESCC (interaction *P*=0.0065), the association between serum 25(OH)D concentration and risk of ESCC only being evident in men. In men we found a clear monotonically increasing risk of ESCC, and subjects in the fourth quartile had a 77% higher risk of ESCC than subjects in the lowest quartile (HR (95% CI)=1.77 (1.16–2.70)). This significant association was present for the continuous variable, for the quartiles, and for the quartile trend test. For ESCC in men, and using cut points of (<40, 40–75, 75+) we found that compared to subjects with serum 25(OH)D of less than 40 nmol l^−1^ subjects in the middle and upper category were associated with increased risk of cancer with HR (95% CI) of 1.15 (0.82–1.61) and 2.01 (1.33–3.05), respectively.

To characterise better the association between serum 25(OH)D concentration and risk of ESCC in men, we tested for deviations from linearity using several methods and found no evidence of nonlinearity. We split the cohort at the concentration of adequacy, 40 nmol l^−1^ and found no evidence of a difference in the slope of the continuous variable below and above 40 nmol l^−1^ (*P*=0.47), and the continuous estimates were similar. Below 40 nmol l^−1^ the HR (95% CI) for continuous vitamin D scaled to 15 nmol l^−1^ was 1.27 (0.97–1.66), and above 40 nmol l^−1^ it was 1.18 (1.07–1.30).

Finally, we explored several other potentially confounding variables. We separately added variables for body mass index (BMI), serum selenium, cholesterol and retinol, and cholesterol and *α*-tocopherol and found that the serum 25(OH)D associations were independent of these exposures and that there was no evidence of interaction (data not shown).

## DISCUSSION

Contrary to previous reports, which examined indirect measures of vitamin D status (Launoy *et al*, 1998; Grant, 2002; Giovannucci *et al*, 2006), we found that higher serum 25(OH)D concentration was associated with increased risk of ESCC, but not with cardia or noncardia gastric cancer. This increased risk of ESCC appeared to vary directly with increasing serum concentrations and there was no evidence of thresholds or deviations from linearity. The association was strong in men and absent in women. Unlike low risk areas for ESCC where this disease is much more common in men than in women, in Linxian the incidence rates are only slightly lower in women than in men and sex is not a risk factor for this disease in our cohort (Tran *et al*, 2004). We have previously found little evidence of differences in risk factors for ESCC between men and women in our cohort. Our previous findings that higher serum concentrations of selenium (Mark *et al*, 2000) and *α*-tocopherol (Taylor *et al*, 2003) were associated with lower risk of ESCC and gastric cardia adenocarcinoma did not differ by sex.

Previous reports that vitamin D was associated with reduced risk of oesophageal cancer did not use serum 25(OH)D to assess status. A French case–control study used estimated dietary vitamin D and found an OR of 0.70 for ESCC for subjects in the highest quartile of estimated vitamin D intake compared to those in the lowest (Launoy *et al*, 1998). This method does not account for UVB generated vitamin D, the primary vitamin D source for most people. An ecologic study of UVB radiation and oesophageal and stomach cancer in the United States found negative correlations of similar magnitude between UVB exposure and oesophageal or stomach cancer rates (Grant, 2002). This study is subject to all the limitations attendant to ecologic studies. Finally, a third study used predicted vitamin D status based on individual subject's race, geographic region of residence, estimated dietary vitamin D, BMI, and physical activity that had *r*^2^=0.28 with measured serum 25(OH)D concentration (Giovannucci *et al*, 2006). This study from the United States reported significant RR (95%, CI) of 0.37 (0.17–0.80) and 0.58 (0.26–1.33) for oesophageal and stomach cancer, respectively, for a 25 nmol l^−1^ increase in predicted serum 25(OH)D status. These authors did not distinguish between ESCC and oesophageal adenocarcinoma, but this seems unlikely to explain the difference between their results and ours since they reported lower risk of most digestive tract cancers regardless of histology.

We note that two previous reports showed that higher measured serum 25(OH)D concentrations are associated with increased cancer risk in men. In a cohort of men from Nordic countries, a U-shaped association for prostate cancer was shown, such that a serum 25(OH)D concentration ⩾80 nmol l^−1^ was associated with at 70% higher risk of prostate cancer than with a concentration between 40 and 59 nmol l^−1^ (Tuohimaa *et al*, 2004). In that cohort, men with serum concentrations ⩽19 nmol l^−1^ were at non-significantly higher risk than those in the reference range. Second, an increasing risk of pancreatic cancer was found with increasing serum 25(OH)D concentration in the all male ATBC cohort from Finland (Stolzenberg–Solomon *et al*, 2006). Men with a serum concentration >65.5 nmol l^−1^ were at threefold higher risk of pancreatic cancer than men with a serum concentration <32 nmol l^−1^. All three studies showing higher risk of cancer associated with higher serum vitamin D status found this association in men. One was restricted to men because it was prostate cancer, one because it was only studied in men, and ours because the association was restricted to men. No simple rationale for a difference in the association by sex is evident and whether there is a consistent difference will require future studies. At least one study found a significant protective effect of higher serum 25(OH)D concentration on risk of colorectal cancer in women (Feskanich *et al*, 2004).

Both of the previous studies, which showed higher risk of cancer with higher serum 25(OH)D concentration, were in Nordic countries that, like our population have relatively low population mean serum 25(OH)D concentrations. Our blood samples were drawn in March when the population mean was probably at or near its nadir, which may not reflect population rank at other times of the year. About 60% of our population had a serum 25(OH)D concentration <40 nmol l^−1^ and nearly the entire cohort was below 80 nmol l^−1^, the concentration that may be necessary to maintain optimal calcium homoeostasis (Zittermann, 2003). The mean serum concentration in US white males in the NHANES III study was 83 nmol l^−1^ (Zadshir *et al*, 2005), but this was not corrected for season and probably represents an average of samples collected throughout the year. We cannot speculate on the association between serum 25(OH)D concentration and risk of ESCC or gastric cancer at the higher serum concentrations seen in the United States and many other countries and it is possible that subjects in the normal to high range of serum vitamin D values may show differences.

An association between higher serum 25(OH)D concentration and increased risk of prostate cancer could be due to the correlation with higher retinol status, which may have adverse effects on prostate cancer (Tuohimaa *et al*, 2004). Our population generally has low serum concentrations of both nutrients, retinol is not associated with ESCC risk in our cohort (Abnet *et al*, 2003), and controlling for serum retinol did not affect the hazard estimates for serum 25(OH)D concentration. One proposed mechanism of vitamin D cancer prevention is that it suppresses high-fat diet induced epithelial cell hyperproliferation (Xue *et al*, 1999). The subjects in this study do not consume high fat diets so the relevance of these findings is limited. A recent report demonstrated that the active vitamin D metabolite 1,25-dihydroxyvitamin D_3_ can induce phase I and phase II metabolising enzymes in the intestine of vitamin D-deficient rats (Kutuzova and Deluca, 2007). This cohort is vitamin D deficient and highly exposed to polycyclic aromatic hydrocarbons (Roth *et al*, 1998a, 1998b), due to using coal for cooking and heating. The induction of phase I metabolising genes may lead to increased activation to reactive intermediates of these pro-carcinogens, thereby increasing cancer risk (Shimada, 2006).

As in all observational epidemiologic studies, we cannot confirm that the association between higher serum 25(OH)D and risk of ESCC in men is causal and not due to confounding by other unmeasured factors.

In conclusion, we found a direct association between higher serum 25(OH)D concentration and increased risk of ESCC in men but not women in a large population-based prospective cohort study from rural China. We found no association with risk of gastric cardia or noncardia adenocarcinoma in either sex. Greater than 50% of our cohort had an inadequate serum 25(OH)D concentration, yet higher concentrations were associated with increased risk of ESCC compared to lower concentrations.

## Figures and Tables

**Table 1:** Nutrition intervention trial case–cohort subject characteristics by case status

**Variable**	**Subcohort^a^**	**ESCC**	**Gastric cardia**	**Gastric noncardia**
Total no.	1105	545	353	81
Men, *N* (%)	610 (55%)	261 (48%)	204 (58%)	60 (74%)
Women, *N* (%)	495 (45%)	284 (52%)	149 (42%)	21 (26%)
				
Age, mean (s.d.)	56.6 (7.9)	56.4 (8.1)	57.1 (7.1)	58.5 (7.2)
Men, mean (s.d.)	57.7 (7.4)	57.4 (7.6)	57.8 (6.9)	59.7 (6.7)
Women, mean (s.d.)	55.3 (8.3)	55.4 (8.5)	56.1 (7.3)	55.1 (7.8)
				
Smoke, *N* (%) yes	427 (39%)	204 (37%)	144 (41%)	41 (51%)
Men, *N* (%) yes	425 (70%)	204 (78%)	144 (71%)	41 (68%)
Women, *N* (%) yes	2 (0.4%)	0 (0%)	0 (0%)	0 (0%)
				
Drink, *N* (%) yes	230 (21%)	123 (23%)	77 (22%)	20 (25%)
Men, *N* (%) yes	201 (33%)	105 (40%)	64 (31%)	19 (31%)
Women, *N* (%) yes	29 (6%)	18 (6%)	13 (9%)	1 (5%)

a Some subjects who later developed one of these cancers are included in the randomly selected subcohort. Therefore, the total number of subjects in this table is greater than the 2018 with measured serum 25(OH)D concentrations.

**Table 2:** Serum 25(OH)D_3_ concentration geometric means (nmol l^−1^) and selected quantiles overall and by sex in the NIT General Population Trial cohort and in cancer cases

			**Quantile**		
	***N***	**Geometric mean^a^**	**25th**	**50th**	**75th**
NIT General Population Trial Cohort^b^					
Overall	2018	31.7	19.6	31.9	48.7
					
Men, overall	1090	33.5	20.3	33.6	51.6
Age strata 1 (40–50)	170	29.5	18.4	30.4	43.7
Age strata 2 (>50–60)	497	36.7	24.2	38.5	59.2
Age strata 3 (>60–70)	423	37.1	23.3	37.5	58.2
Never smokers	309	33.6	19.5	36.1	60.3
Ever smokers^c^	781	33.4	21.2	32.9	48.9
Non-drinkers	717	34.7	21.4	35.7	53.7
Drinkers^d^	373	31.2	18.6	32.2	47.4
					
Women^e^, overall	928	30.4	18.7	30.7	45.9
Age strata 1 (40–50)	244	31.9	20.1	31.7	47.3
Age strata 2 (>50–60)	402	27.9	17.7	28.7	42.2
Age strata 3 (>60–70)	282	31.5	19.6	28.9	50.6
					
*Cancer cases*					
ESCC	545	33.7	19.9	33.4	57.2
Gastric cardia adenocarcinoma	353	33.6	19.2	33.8	54.5
Gastric noncardia adenocarcinoma	81	33.5	19.1	37.5	56.1

Abbreviation: ESCC=oesophageal squamous cell carcinomas.

a Serum vitamin D distributions were skewed and normality was improved by log transformation, so geometric means are presented.

b All concentrations are weighted by the age and sex sampling to reflect the distributions in the full cohort.

c Daily cigarette consumption among men was generally low and few subjects had quit smoking, so smoking is presented as never smoking *vs* ever smoking.

d Alcohol consumption was minimal at the time of the baseline interview (1985), so alcohol drinking was categorized as any consumption in the previous 12 months *vs* none.

e Cigarette and alcohol consumption were mainly restricted to men (see Table 1), so stratifications are not presented for women.

**Table 3:** Adjusted^a^ hazard ratios (HR) and 95% confidence intervals (CI) for the association between serum 25(OH)D_3_ concentration and risk of ESCC, gastric cardia adenocarcinoma, and gastric noncardia adenocarcinoma in the NIT General Population Trial cohort (1986–1991)

	**Continuous^b^**			**Quartile**							
				**Q1**	**Q2**		**Q3**		**Q4**		
**Group**	**HR**	**95% CI**	***P***	**Ref**	**HR**	**95% CI**	**HR**	**95% CI**	**HR**	**95% CI**	***P*_trend_ c**
*ESCC*											
Overall^d^, *N* cases/controls^e^	545 : 1071			142 : 267	123 : 267		116 : 269		164 : 268		
	1.06	1.01–1.13	0.026	1.0	0.87	0.64–1.17	0.85	0.63–1.16	1.30	0.97–1.73	0.013
Men, *N* cases/controls	261 : 590			55 : 149	56 : 148		67 : 147		83 : 146		
	1.15	1.07–1.25	0.00013	1.0	1.04	0.66–1.61	1.31	0.85–2.00	1.77	1.16–2.70	0.0033
Women, *N* cases/controls	284 : 481			78 : 120	79 : 118		47 : 122		80 : 121		
	0.98	0.90–1.07	0.71	1.0	1.00	0.67–1.49	0.60	0.38–0.93	1.06	0.71–1.59	0.70
											
*Gastric cardia adenocarcinoma*											
Overall^d^, *N* cases/controls	353 : 1086			97 : 271	73 : 268		80 : 273		103 : 274		
	1.03	0.96–1.10	0.36	1.0	0.77	0.54–1.09	0.81	0.57–1.15	1.11	0.80–1.55	0.49
Men, *N* cases/controls	204 : 597			54 : 148	43 : 149		49 : 150		58 : 150		
	1.06	0.97–1.15	0.22	1.0	0.81	0.51–1.29	0.91	0.58–1.42	1.14	0.73–1.77	0.51
Women, *N* cases/controls	149 : 489			37 : 123	33 : 122		42 : 121		37 : 123		
	0.99	0.89–1.11	0.92	1.0	0.92	0.54–1.57	1.17	0.70–1.97	1.09	0.64–1.85	0.55
											
*Gastric noncardia adenocarcinoma*											
Overall^d^, *N* cases/controls	81 : 1096			22 : 274	10 : 276		23 : 274		26 : 272		
	0.98	0.86–1.12	0.77	1.0	0.46	0.21–0.99	0.95	0.51–1.76	1.10	0.60–2.01	0.39
Men, *N* cases/controls	60 : 601			18 : 150	7 : 153		21 : 149		14 : 149		
	0.94	0.80–1.09	0.40	1.0	0.40	0.16–0.98	1.17	0.60–2.29	0.80	0.38–1.67	0.87
Women, *N* cases/controls	21 : 495			4 : 124	5 : 124		6 : 124		6 : 123		
	1.11	0.89–1.38	0.37	1.0	1.39	0.36–5.36	1.66	0.45–6.12	1.29	0.46–3.22	0.40

Abbreviations: CI=confidence interval; ESCC=oesophageal squamous cell carcinomas; HR=hazard ratio.

a HR and 95% CI in the overall models were calculated using models stratified on age and sex with additional adjustment by separate continuous age variables for each stratum, and for cigarette smoking and alcohol drinking. Models in women were not adjusted for smoking because <1% of women smoked cigarettes.

b Continuous HRs are scaled to 15 nmol l^−1^, the approximate size of one central quartile in the overall population distribution given in Table 2.

c *P* for trend comes from a model where quartile of 25(OH)D status was entered as an ordinal variable.

d P for interaction between serum vitamin D concentration (continuous variable) and sex: ESCC=0.0065; Gastric cardia adenocarcinoma, *P*=0.44; Gastric noncardia adenocarcinoma, *P*=0.21.

e This study used a case–cohort design, therefore control numbers are slightly lower than the subcohort number presented in Table 1.
